# Supplementary material for: Pleiotropic mutational effects on function and stability constrain the antigenic evolution of influenza hemagglutinin
Source: bioRxiv. 2025 Sep 30:2025.05.24.655919. Originally published 2025 May 24. Preprint. [Version 2] doi: 10.1101/2025.05.24.655919 (PMC12139742; doi:10.1101/2025.05.24.655919)
Supplement: 1 [file NIHPP2025.05.24.655919v2-supplement-1.pdf]

bioRxiv preprint doi: https://doi.org/10.1101/2025.05.24.655919; this version posted September 30, 2025. The copyright holder for this preprint (which was not certified by peer review) is the author/funder, who has granted bioRxiv a license to display the preprint in perpetuity. It is made available under aCC-BY 4.0 International license.

Supplemental figures

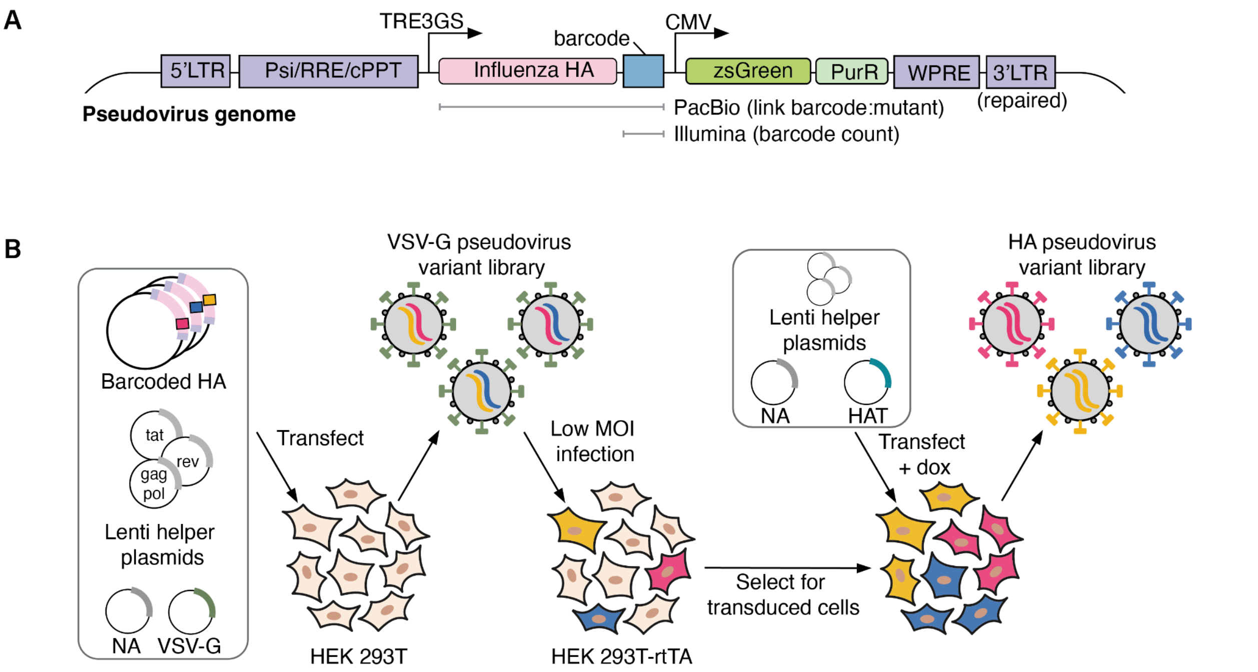

C

Library	Number of barcodes	% mutations present
Library A	64032	98.7
Library B	70581	99.0

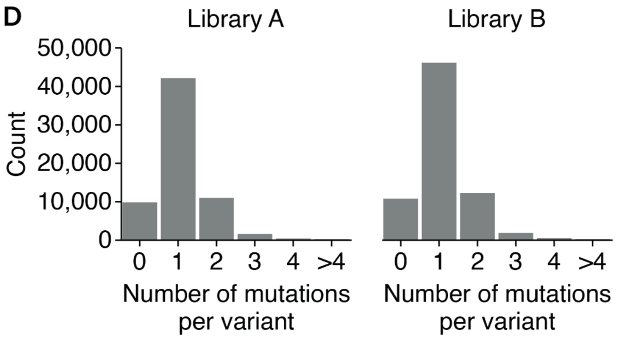

Extended Data Figure 1 | Pseudovirus deep mutational scanning of influenza hemagglutinin.

A) Diagram of the lentiviral genome used to produce genotype-phenotype-linked pseudovirus libraries for deep mutational scanning. The genome is flanked by long terminal repeat (LTR) sequences, with the typical 3′ LTR deletion repaired so the lentiviral genome can be transcribed after integration. A zsGreen reporter and a puromycin resistance marker are constitutively expressed from a CMV promoter. Expression of the HA gene is regulated by a doxycycline inducible TRE3GS promoter. PacBio sequencing is performed to map each barcode to an HA mutant. Then, effects of HA mutations can be quantified by Illumina sequencing the barcodes. **B)** Schematic of the "two-step" method for generating genotype-phenotype-linked pseudovirus libraries described in Dadonaite et al.[23,24]. In the first step, a plasmid library encoding the lentiviral genomes with the HA mutants is co-transfected into HEK293T cells alongside three lentiviral helper plasmids (tat, rev, gagpol), a plasmid expressing a strain-matched neuraminidase (NA), and a plasmid expressing the glycoprotein from vesicular stomatitis virus

bioRxiv preprint doi: https://doi.org/10.1101/2025.05.24.655919; this version posted September 30, 2025. The copyright holder for this preprint (which was not certified by peer review) is the author/funder, who has granted bioRxiv a license to display the preprint in perpetuity. It is made available under aCC-BY 4.0 International license.

(VSV-G). This results in pseudoviruses that encode HA mutants within their genomes but express VSV-G and NA on their surfaces. The NA ensures HA expression does not prevent virions from detaching from producing cells. These VSV-G pseudotyped viruses are transduced into a HEK293T-rtTA cell line at low MOI to ensure most infected cells integrate a single lentiviral genome, and puromycin is used to select for integrated cells. In the second step, helper plasmids, a plasmid expressing NA, and a plasmid expressing the HA-activating human airway trypsin-like (HAT) protease are co-transfected into the integrated cells. Doxycycline is added at this step to induce HA expression. This results in genotype-phenotype-linked HA-pseudotyped pseudoviruses. These pseudoviruses can undergo a single round of cell entry, but are not fully infectious agents as they do not encode the genes needed to undergo multiple rounds of replication. **C)** Number of barcodes and mutation coverage in the two pseudovirus library replicates. In this table, "% mutations present" indicates the percentage of all HA ectodomain amino-acid mutations found in at least one of the barcoded variants. **D)** Distribution of the number of HA amino-acid mutations per variant in the two pseudovirus library replicates. Most variants contain a single mutation.

bioRxiv preprint doi: https://doi.org/10.1101/2025.05.24.655919; this version posted September 30, 2025. The copyright holder for this preprint (which was not certified by peer review) is the author/funder, who has granted bioRxiv a license to display the preprint in perpetuity. It is made available under aCC-BY 4.0 International license.

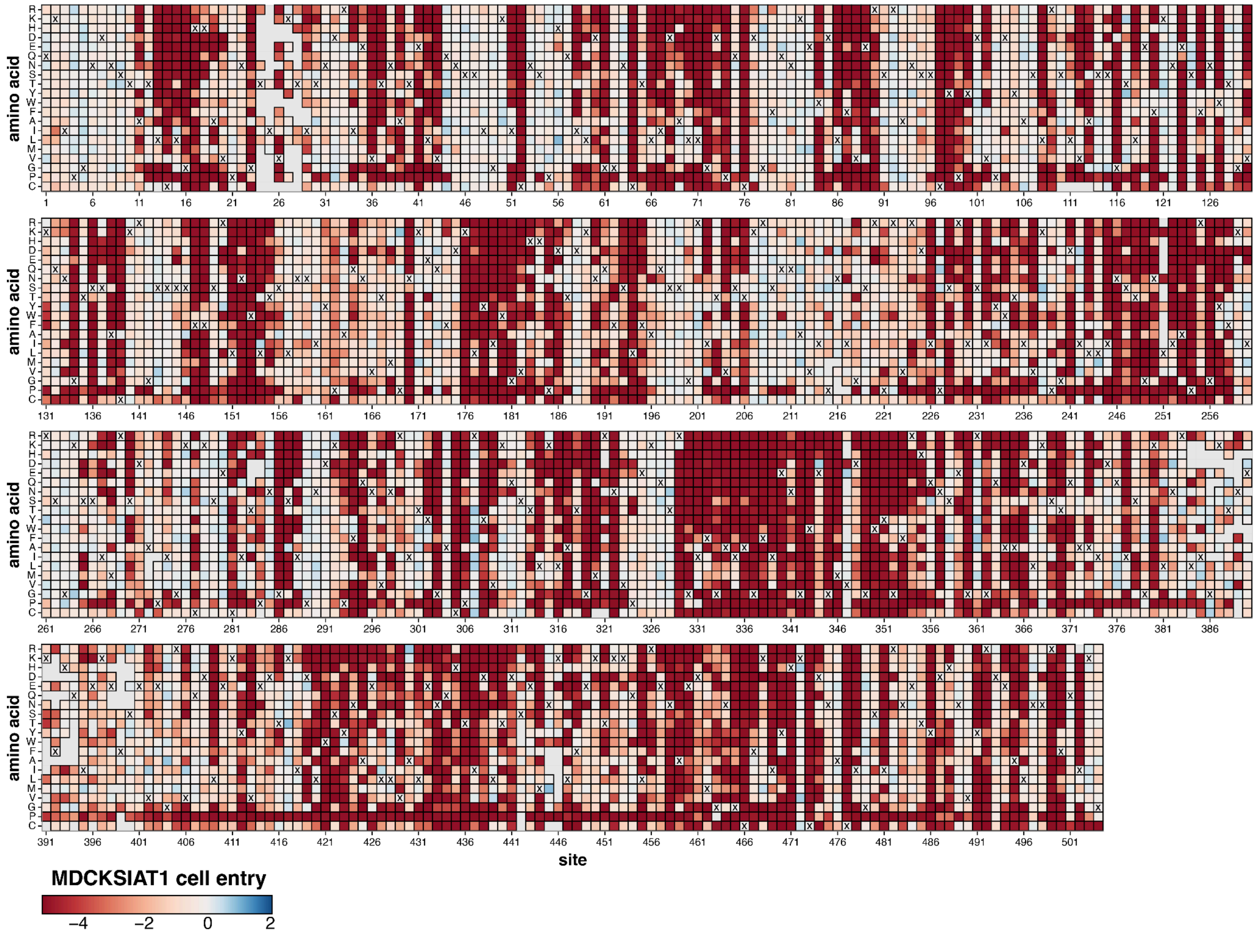

Extended Data Figure 2 | Mutation effects on HA-mediated cell entry.

Each tile represents a mutation at an HA site, colored by the effect of that mutation on entry into MDCK-SIAT1 cells. Red indicates impaired entry, white indicates no effect, and blue indicates improved entry. To visualize these mutation effects in the context of the HA structure, see **Fig. 1B**. Tiles with an 'X' denote the amino acid identity in the unmutated MA22 strain. Empty gray tiles indicate mutations that were either missing from the library or lacked a reliable cell entry measurement. See https://dms-vep.org/Flu_H3_Massachusetts2022_DMS/cell_entry.html for an interactive version of this heatmap.

bioRxiv preprint doi: https://doi.org/10.1101/2025.05.24.655919; this version posted September 30, 2025. The copyright holder for this preprint (which was not certified by peer review) is the author/funder, who has granted bioRxiv a license to display the preprint in perpetuity. It is made available under aCC-BY 4.0 International license.

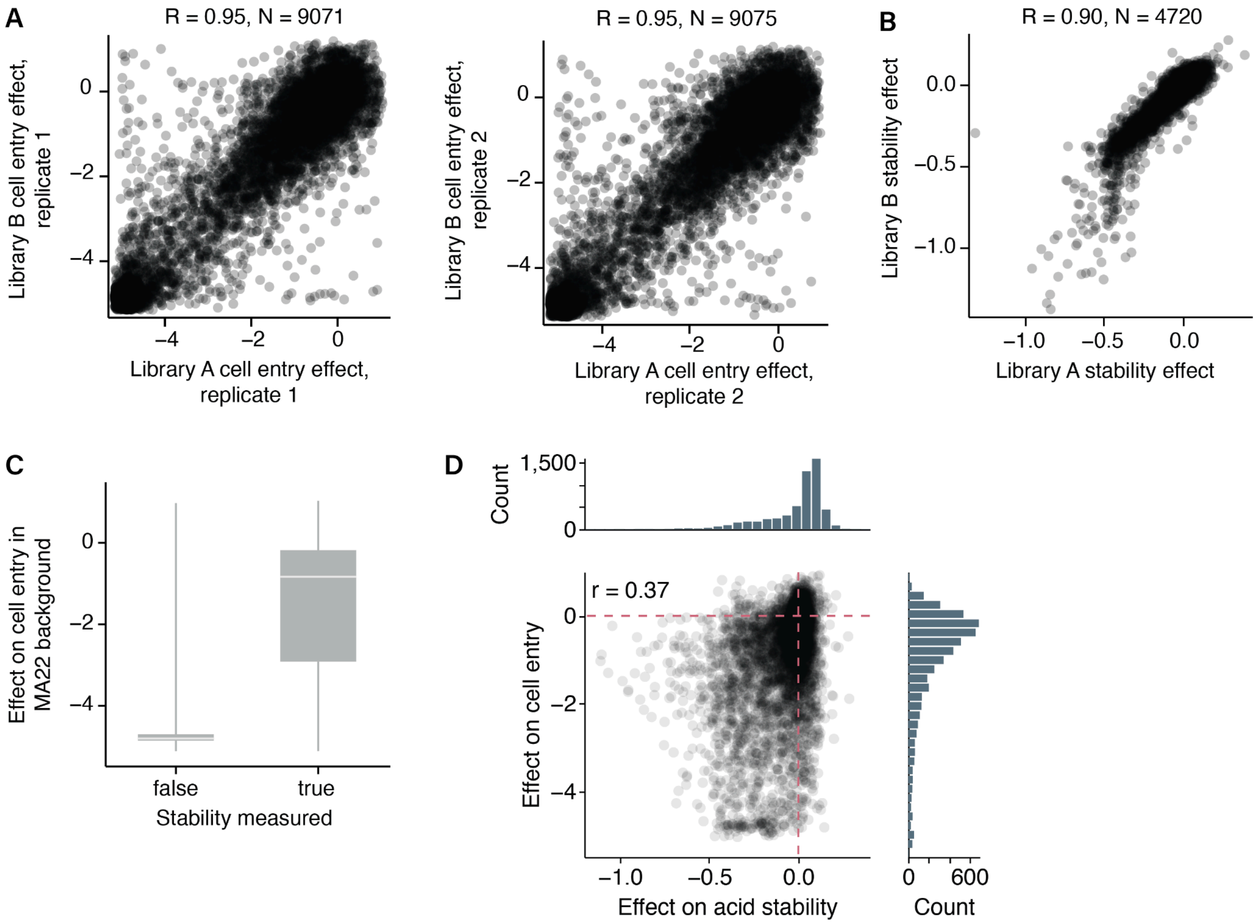

Extended Data Figure 3 | Correlations of mutation effects measured by deep mutational scanning.

A) Correlation of the effects of HA mutations on cell entry between the two pseudovirus library replicates. Each point represents the effect of a different mutation as measured in each replicate. Two technical replicates were performed for each of the two libraries, and the two panels show correlations between the two independent libraries for each technical replicate. Throughout this paper, we report the median effect of mutations across the four replicates. **B)** Correlation of the effects of HA mutations on acid stability between the two pseudovirus library replicates. Note that there are fewer mutations with measured effects on stability because we can only measure stability for mutations with at least some cell entry. **C)** Most mutations for which it was not possible to make measurements of acid stability correspondingly have very poor cell entry. The center line shows the median effect on cell entry, the box indicates the interquartile range, and the whiskers extend 1.5 x interquartile range beyond the first and third quartiles. **D)** Correlation between effects of mutations on cell entry and effects of mutations on acid stability. These effects of mutations on these two phenotypes are only weakly correlated.

bioRxiv preprint doi: https://doi.org/10.1101/2025.05.24.655919; this version posted September 30, 2025. The copyright holder for this preprint (which was not certified by peer review) is the author/funder, who has granted bioRxiv a license to display the preprint in perpetuity. It is made available under aCC-BY 4.0 International license.

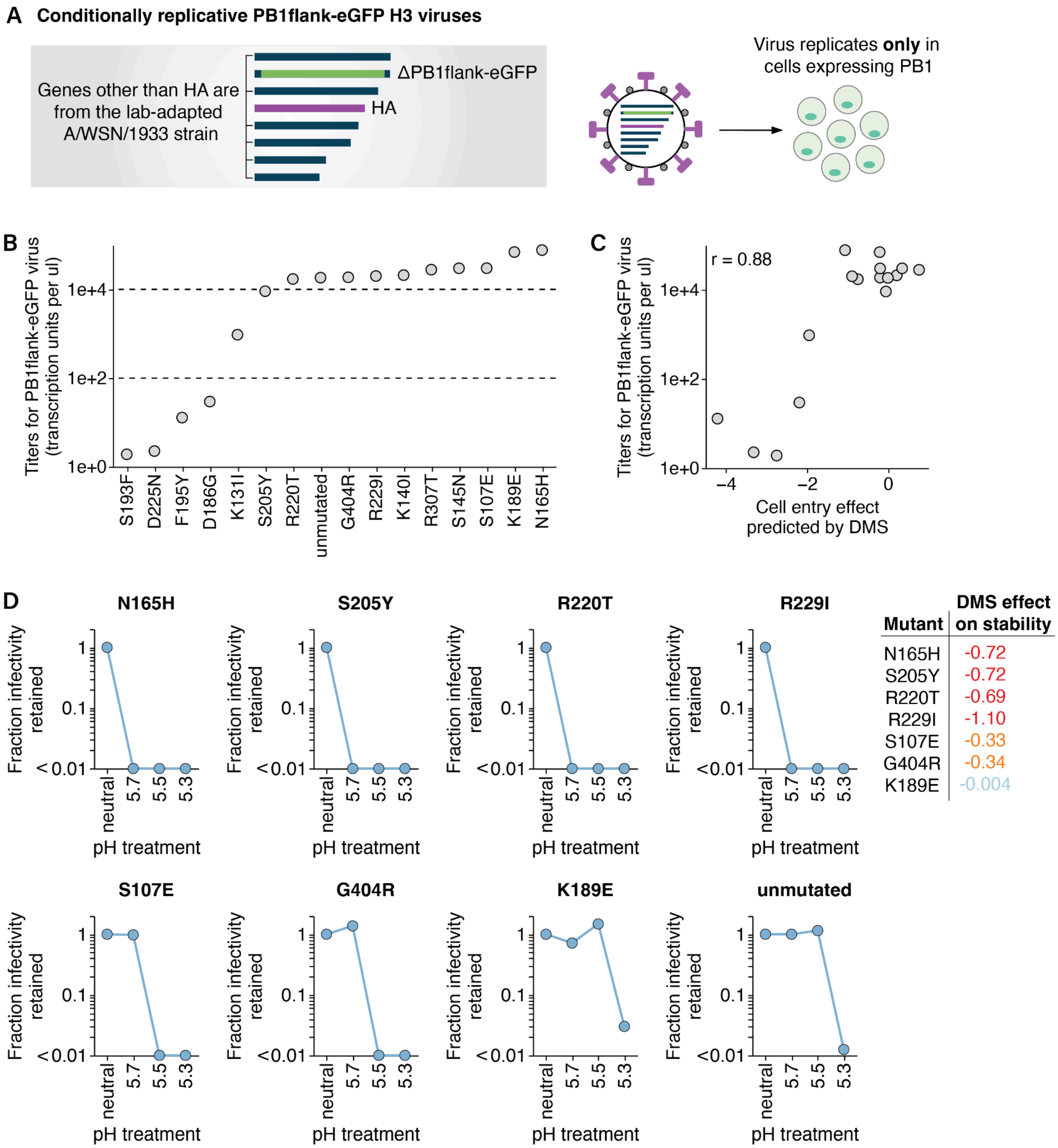

Extended Data Figure 4 | Validation of mutation effects on cell entry and acid stability with conditionally replicative influenza virions.

A) Diagram of a conditionally replicative PB1flank-eGFP influenza virus genome. The PB1 gene is replaced with an eGFP and the remaining segments besides HA are derived from a lab-adapted A/WSN/1933 strain. These viruses can be rescued by reverse genetics, can only replicate in cells that express PB1, and are safe to use at biosafety-level 2. **B)** Titers of

bioRxiv preprint doi: https://doi.org/10.1101/2025.05.24.655919; this version posted September 30, 2025. The copyright holder for this preprint (which was not certified by peer review) is the author/funder, who has granted bioRxiv a license to display the preprint in perpetuity. It is made available under aCC-BY 4.0 International license.

conditionally replicative virions carrying single amino acid mutations in the MA22 HA that were present in the supernatant after virus production by reverse genetics. Each point in the plot is the mean of four titer measurements, two technical replicates from the same virion rescue stock and two biological replicate stocks rescued from independent plasmid preparations. **C)** Correlation between the titers of conditionally replicative virions carrying single amino acid mutations to the MA22 HA (shown in B) and the effects of those mutations on cell entry measured by deep mutational scanning. **D)** The fraction infectivity retained after treating conditionally replicative virions with the indicated MA22 HA mutations with either neutral media or acidic pH buffers. The fractions are normalized to the infectivity in the neutral condition. Each point is the mean of two technical replicates performed on different days. The effect of each mutant on acid stability measured by deep mutational scanning is included on the right, and generally tracks with the pH sensitivity measured in the validation assay.

bioRxiv preprint doi: https://doi.org/10.1101/2025.05.24.655919; this version posted September 30, 2025. The copyright holder for this preprint (which was not certified by peer review) is the author/funder, who has granted bioRxiv a license to display the preprint in perpetuity. It is made available under aCC-BY 4.0 International license.

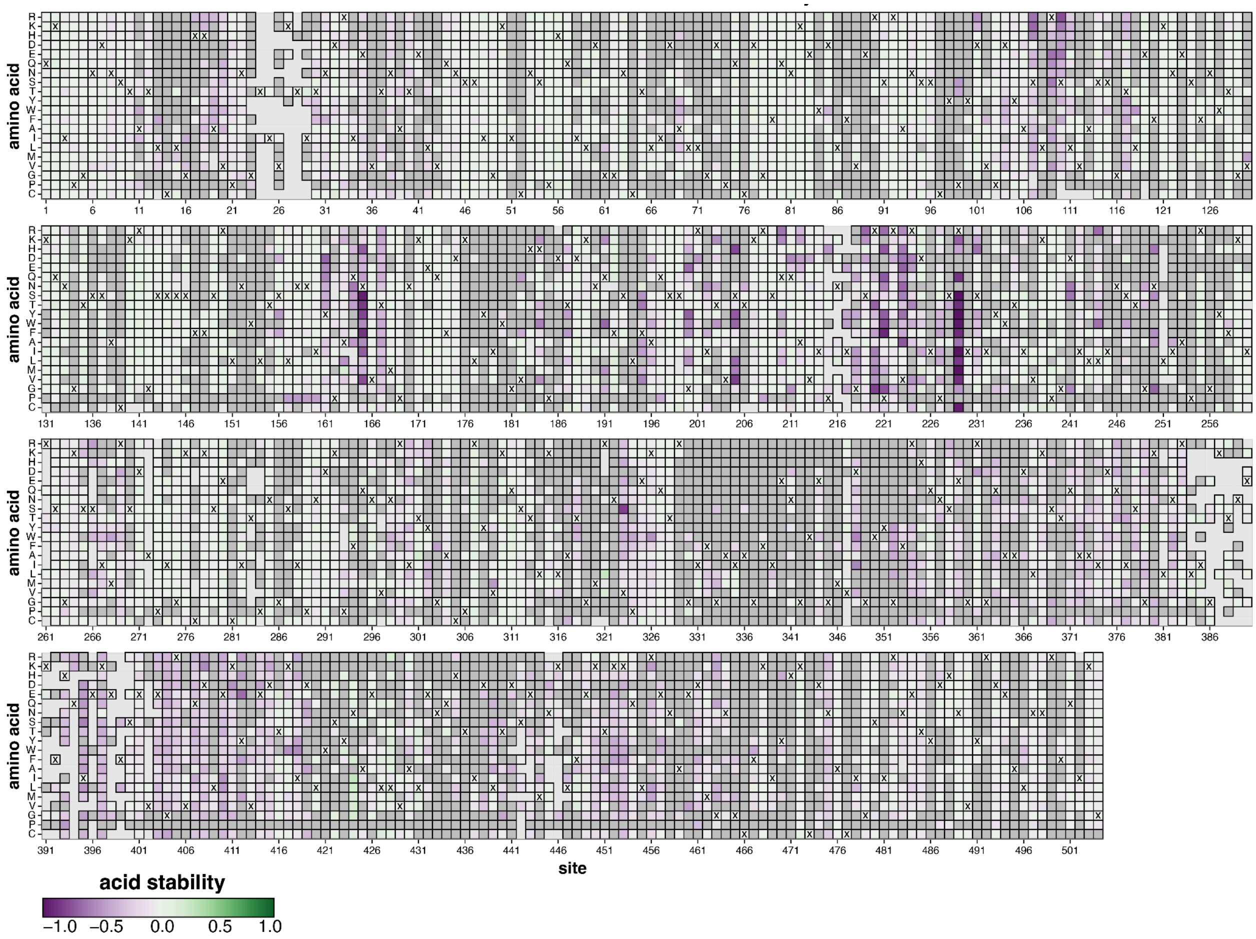

Extended Data Figure 5 | Mutation effects on HA acid stability.

Each tile represents a mutation at an HA site, colored by the effect of that mutation on HA acid stability. Purple indicates decreased stability, white indicates no effect, and green indicates increased stability. To visualize these mutation effects in the context of the HA structure, see **Fig. 2C**. Tiles with an 'X' denote the amino acid identity in the unmutated MA22 strain. Dark gray tiles indicate mutations that are too deleterious for cell entry to reliably measure their effect on acid stability, while light gray tiles indicate mutations that were missing (not measured) in the library. See https://dms-vep.org/Flu_H3_Massachusetts2022_DMS/acid_stability.html for an interactive version of this heatmap.

bioRxiv preprint doi: https://doi.org/10.1101/2025.05.24.655919; this version posted September 30, 2025. The copyright holder for this preprint (which was not certified by peer review) is the author/funder, who has granted bioRxiv a license to display the preprint in perpetuity. It is made available under aCC-BY 4.0 International license.

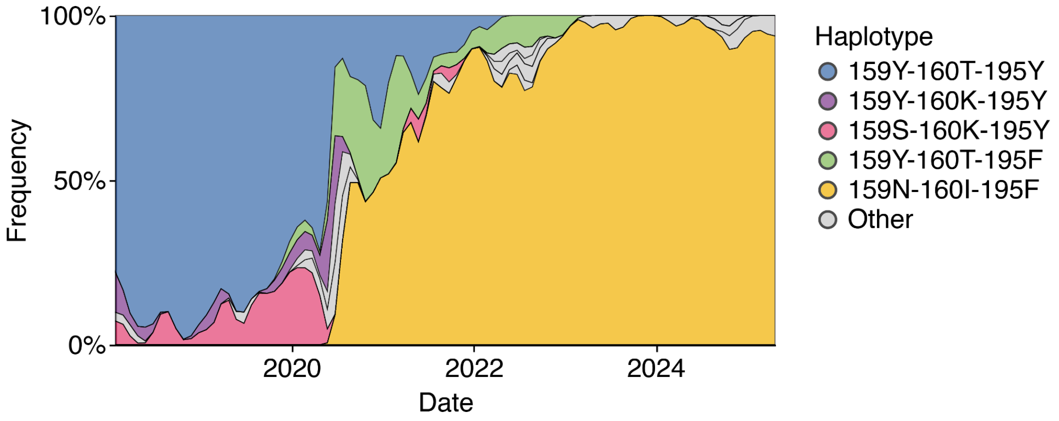

Extended Data Figure 6 | Evolutionary dynamics at sites 159, 160, and 195 in human H3N2 HA.

Muller diagram showing all combinations of amino acids observed at sites 159, 160, and 195 since 2018 in the evolution of the HA from human H3N2 influenza. Haplotypes that reach a frequency >20% at some timepoint are colored according to the key, while other haplotypes are colored gray. Y159N and T160I only arise to fixation in the background of 195F, and this triple mutant lineage (yellow) eventually outcompetes the lineage containing 195F alone (light green).

bioRxiv preprint doi: https://doi.org/10.1101/2025.05.24.655919; this version posted September 30, 2025. The copyright holder for this preprint (which was not certified by peer review) is the author/funder, who has granted bioRxiv a license to display the preprint in perpetuity. It is made available under aCC-BY 4.0 International license.

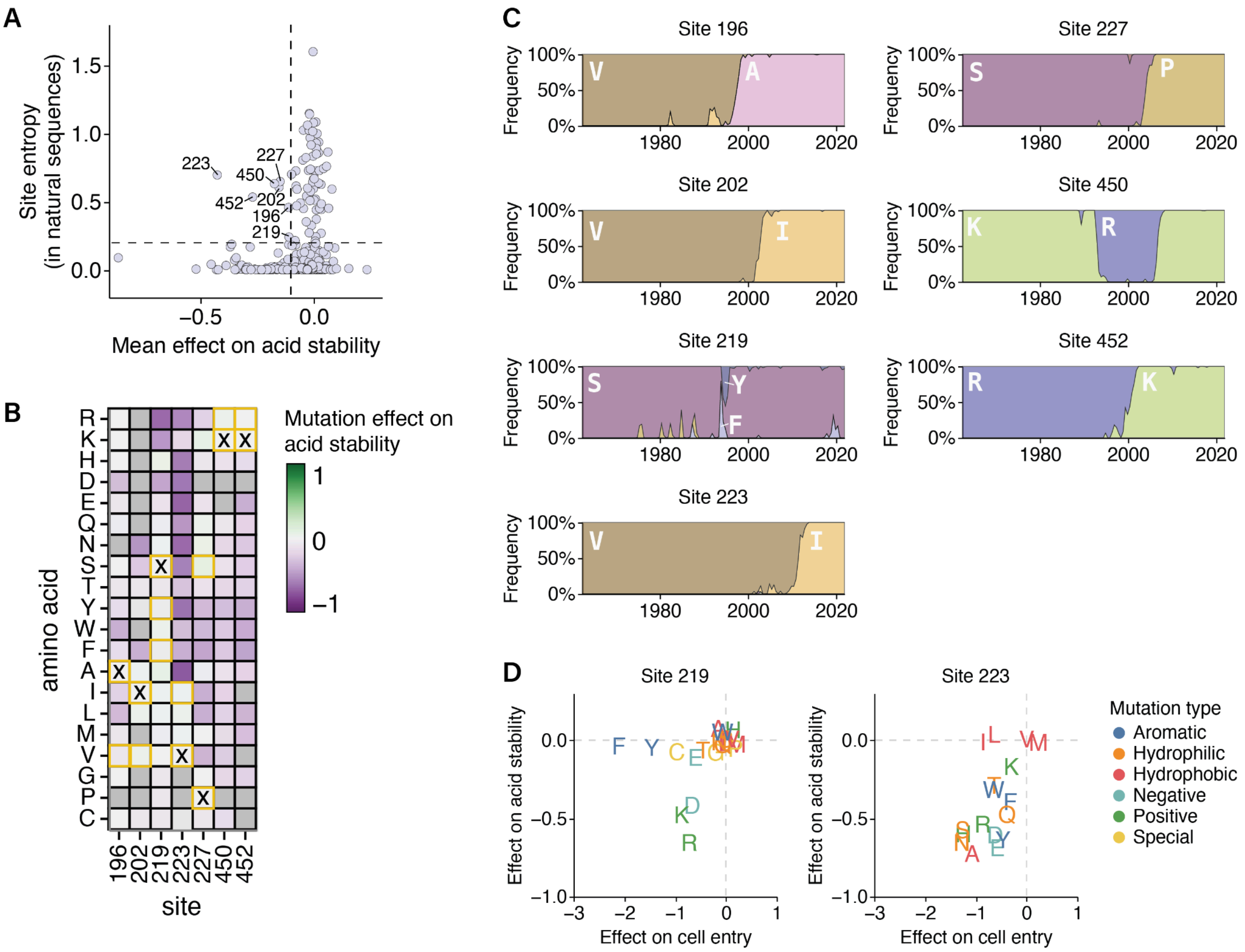

Extended Data Figure 7 | Conservation at HA sites with destabilizing mutations.

A) Correlation between the Shannon entropy at each HA site in an alignment from a subsampled tree of natural human H3N2 evolution since 1968 and the mean effect on acid stability of all mutations at each HA site as measured by deep mutational scanning. Most sites with many mutations that destabilize HA are strongly conserved, with exceptions labeled. **B)** Heatmap of all mutation effects on acid stability at sites labeled in A. The 'X' indicates the amino acid in the unmutated MA22 HA, and squares boxed in yellow indicate amino acids that increased in frequency during natural evolution. At sites with many destabilizing mutations, natural evolution exclusively samples only the (relatively rare) amino-acid identities that do not destabilize HA. **C)** Frequencies over time (the x-axis indicates year) of amino acids observed in natural human H3N2 sequences at the sites labeled in A. **D)** Correlation between mutation effects on acid stability and cell entry at sites 219 and 223. Mutations are colored by biochemical group.

bioRxiv preprint doi: https://doi.org/10.1101/2025.05.24.655919; this version posted September 30, 2025. The copyright holder for this preprint (which was not certified by peer review) is the author/funder, who has granted bioRxiv a license to display the preprint in perpetuity. It is made available under aCC-BY 4.0 International license.

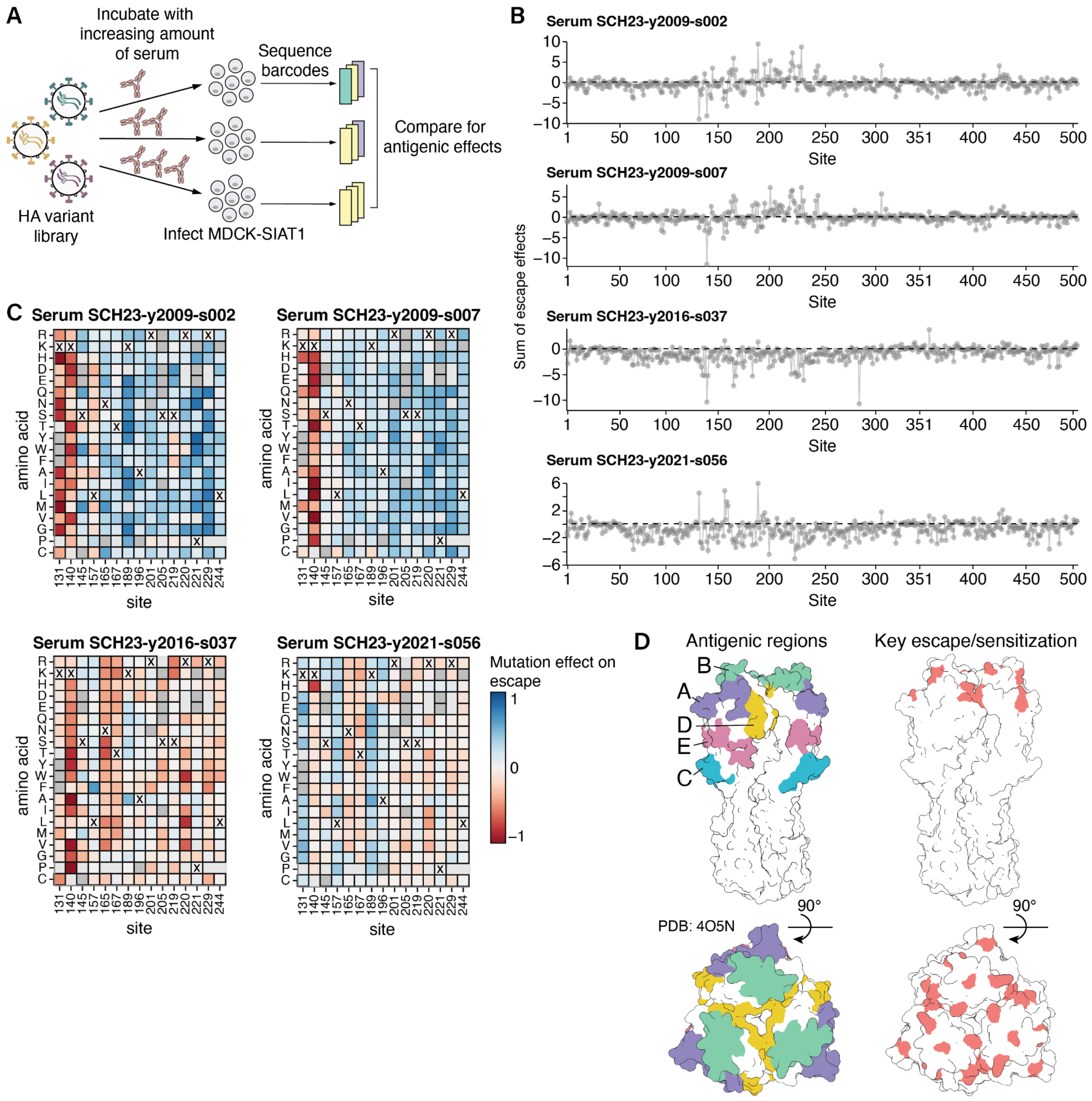

Extended Data Figure 8 | Mapping the effects of HA mutations on neutralization by human sera using deep mutational scanning.

A) We incubated the pseudovirus HA library with increasing concentrations of serum, then infected MDCK-SIAT1 cells and sequenced the barcodes of pseudoviruses that were still able to enter cells after serum treatment. To quantify mutation effects on antigenicity, we compared these barcode counts to those of pseudoviruses that were not incubated with serum, which served as an infection baseline; a neutralization standard is used to convert these counts into fraction infectivity at each serum concentration (**Methods**)[23,24]. The mutation effects we report are the median of two biological replicates. **B)** The sum of mutation effects on escape at each site

bioRxiv preprint doi: https://doi.org/10.1101/2025.05.24.655919; this version posted September 30, 2025. The copyright holder for this preprint (which was not certified by peer review) is the author/funder, who has granted bioRxiv a license to display the preprint in perpetuity. It is made available under aCC-BY 4.0 International license.

in HA for four human sera. **Fig. 4A** shows these four plots overlaid. See https://dms-vep.org/Flu_H3_Massachusetts2022_DMS/sera_neutralization.html for a version of these lineplots that is interactive, along with interactive heatmaps that show how individual mutations affect sera neutralization. **C)** Mutation-level escape and sensitization at key sites that are highlighted in **Fig. 4A** for the four sera. The X's indicate the amino acid in the unmutated MA22 strain. Tiles that are more blue indicate mutations that escape sera, while tiles that are more red indicate mutations that have a sensitizing effect. **D)** HA structures (Protein Data Bank 4O5N) showing antigenic regions and locations of key sites of escape or sensitization that are highlighted in **Fig. 4A**.

bioRxiv preprint doi: https://doi.org/10.1101/2025.05.24.655919; this version posted September 30, 2025. The copyright holder for this preprint (which was not certified by peer review) is the author/funder, who has granted bioRxiv a license to display the preprint in perpetuity. It is made available under aCC-BY 4.0 International license.

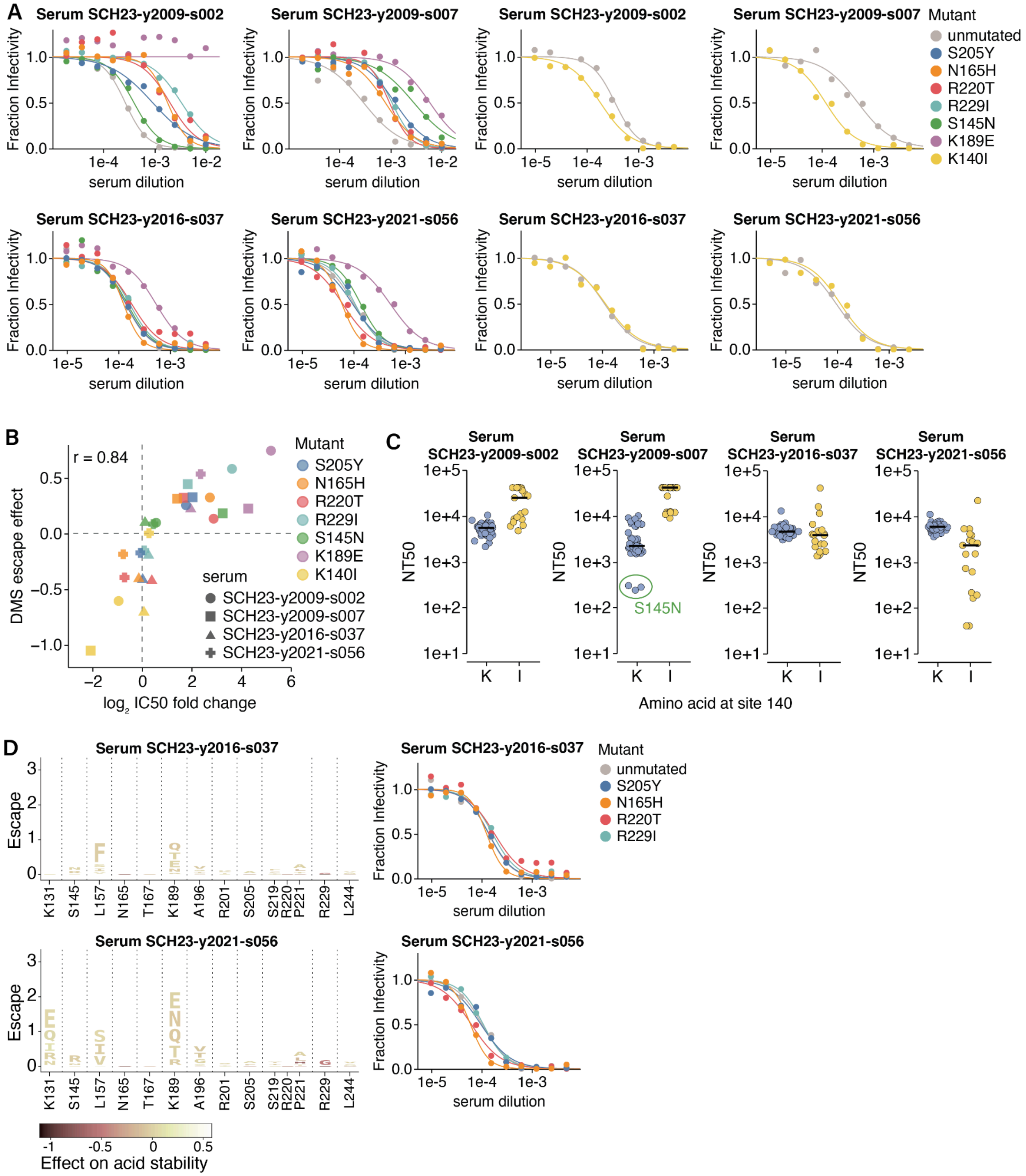

Extended Data Figure 9 | Validation of mutation effects on serum neutralization with conditionally replicative influenza virions.

A) Neutralization of conditionally replicative influenza virions with the MA22 HA carrying the indicated mutations (S205Y, N165H, R220T, R229I, S145N, K189E, and K140I) by four human sera collected in 2023. Each point is the mean of two technical replicates. A subset of these curves are shown in **Fig. 4C**, **Fig. 4F**, and **Extended Data Fig. 9D**. **B)** Correlation between the

bioRxiv preprint doi: https://doi.org/10.1101/2025.05.24.655919; this version posted September 30, 2025. The copyright holder for this preprint (which was not certified by peer review) is the author/funder, who has granted bioRxiv a license to display the preprint in perpetuity. It is made available under aCC-BY 4.0 International license.

antigenic effect of mutations measured by deep mutational scanning and the change in IC50 measured by the independent neutralization assay in A. **C)** Neutralization for 78 vaccine or circulating H3N2 strains between 2012 and 2023 by the four sera, as measured in Kikawa et al.[3]. The points are the median of two or three barcoded replicates and are stratified by whether or not the strain includes a K or I at site 140. The black line indicates the median NT50. Two of the sera have higher titers against strains with 140I, consistent with K140I being a sensitizing mutation for these sera. There are three recent strains that contain S145N that escape SCH23-y2009-s007, consistent with this being an escape mutation from this sera. **D)** Logoplots displaying single nucleotide accessible mutations from MA22 HA with positive escape at the key sites highlighted red in **Fig. 4A** for two sera. The height of each letter is proportional to the escape from the indicated serum as measured by deep mutational scanning. Each mutation is colored by its effect on HA acid stability as measured in the deep mutational scanning, with darker colors indicating decreased stability. The logoplots and neutralization curves for the other two sera mapped by deep mutational scanning are shown in **Fig. 4E** and **Fig. 4F**. In the neutralization curves, each point is the mean of two technical replicates.

bioRxiv preprint doi: https://doi.org/10.1101/2025.05.24.655919; this version posted September 30, 2025. The copyright holder for this preprint (which was not certified by peer review) is the author/funder, who has granted bioRxiv a license to display the preprint in perpetuity. It is made available under aCC-BY 4.0 International license.

Supplemental tables

Extended Data Table 1 | Sites within receptor binding pocket and antigenic regions.

**Receptor binding pocket region**	**Sites**	**Reference**
130-loop	128, 130, 131, 133-138	[10,12,14,75,76]
150-loop	155-160	[10,12,14,75,76]
190-helix	186, 189-194, 196-198	[10,12,14,75,76]
220-loop	221-228	[10,12,14,75,76]
Base	98, 153, 183, 195	[10,12,14,75,76]
Other	145	[77]
**Antigenic region**		
Epitope A	122-146	[33]
Epitope B	155-160, 186-198	[33]
Epitope C	44-54, 273-280	[33]
Epitope D	166-181, 201-219	[33]
Epitope E	62-65, 78-94, 260-265	[33]
